# The dual role of asporin in breast cancer progression

**DOI:** 10.18632/oncotarget.10471

**Published:** 2016-07-07

**Authors:** Dana Simkova, Gvantsa Kharaishvili, Gabriela Korinkova, Tomas Ozdian, Tereza Suchánková-Kleplová, Tomas Soukup, Michal Krupka, Adela Galandakova, Petr Dzubak, Maria Janikova, Jiri Navratil, Zuzana Kahounova, Karel Soucek, Jan Bouchal

**Affiliations:** ^1^ Department of Clinical and Molecular Pathology, Institute of Molecular and Translational Medicine, Faculty of Medicine and Dentistry, Palacky University, Olomouc, Czech Republic; ^2^ Laboratory of Experimental Medicine, Institute of Molecular and Translational Medicine, Faculty of Medicine and Dentistry, Palacky University, Olomouc, Czech Republic; ^3^ Department of Histology and Embryology, Faculty of Medicine in Hradec Kralove, Charles University, Hradec Kralove, Czech Republic; ^4^ Department of Immunology, Faculty of Medicine and Dentistry, Palacky University, Olomouc, Czech Republic; ^5^ Department of Medical Chemistry and Biochemistry, Institute of Molecular and Translational Medicine, Faculty of Medicine and Dentistry, Palacky University, Olomouc, Czech Republic; ^6^ Department of Comprehensive Cancer Care, Masaryk Memorial Cancer Institute, Brno, Czech Republic; ^7^ Department of Cytokinetics, Institute of Biophysics, Academy of Sciences of the Czech Republic, v.v.i., Brno, Czech Republic; ^8^ Center of Biomolecular and Cellular Engineering, International Clinical Research Center, St. Anne's University Hospital Brno, Brno, Czech Republic; ^9^ Department of Experimental Biology, Faculty of Science, Masaryk University, Brno, Czech Republic

**Keywords:** asporin, 3D cultivation, stiffness, grade, breast cancer

## Abstract

Asporin has been reported as a tumor suppressor in breast cancer, while asporin-activated invasion has been described in gastric cancer. According to our in silico search, high asporin expresion associates with significantly better relapse free survival (RFS) in patients with low-grade tumors but RFS is significantly worse in patients with grade 3 tumors. In line with other studies, we have confirmed asporin expression by RNA scope *in situ* hybridization in cancer associated fibroblasts. We have also found asporin expression in the Hs578T breast cancer cell line which we confirmed by quantitative RT-PCR and western blotting. From multiple testing, we found that asporin can be downregulated by bone morphogenetic protein 4 while upregulation may be facilited by serum-free cultivation or by three dimensional growth in stiff Alvetex scaffold. Downregulation by shRNA inhibited invasion of Hs578T as well as of CAFs and T47D cells. Invasion of asporin-negative MDA-MB-231 and BT549 breast cancer cells through collagen type I was enhanced by recombinant asporin. Besides other investigations, large scale analysis of aspartic acid repeat polymorphism will be needed for clarification of the asporin dual role in progression of breast cancer.

## INTRODUCTION

Tumor progression is partly a result of evolving crosstalk between different cell types within the tumor and its surrounding supportive tissue/tumor stroma [1]. The extracellular matrix (ECM) is a dynamic structure providing constructional support, organisation and orientation into tissues [2] as well as supplying crucial elements for cell survival i.e. growth factors, inflammatory molecules and immune soluble mediators [3]. ECM consists of a plethora of proteins of varying structure and function [4]. Inter alia, the small leucine rich proteoglycan family (SLRPs) constitutes an important group of ECM proteins, widely found in most extracellular matrices [5].

Asporin was identified by three research groups in 2001 [6, 7, 8]. The aspartic acid rich N-terminal region and central part of the asporin molecule bind type II collagen [9] while collagen I is bound by the central region. Asporin inhibits collagen fibrilogenesis and competes with decorin in binding the same sites. For this reason, this competition may have a role in regulating the development of ECM. The asporin N-terminal polyaspartate domain also binds calcium, and works in concert with other domains in order to initiate the mineralization of collagen [10]. Asporin plays an important role in normal development, in particular of cartilage, bone and teeth, while its genetic polymorphisms have been associated with various bone and joint diseases, including osteoarthritis, rheumatoid arthritis and lumbar disc disease [11].

We identified asporin by microdissection and expression profiling as a novel breast cancer related protein [12]. It was upregulated in invasive carcinomas, in particular lobular ones, together with other extracellular matrix proteins such as periostin or versican [13, 14]. Asporin can also be found in gene lists from other expression microarray analyses both in breast and other cancer types [15–19]. Association of asporin with different types of cancer has also been confirmed by other methods such as tag profiling and mass spectrometry [20–23]. Important findings have been recently reported by Satoyoshi et al. [24] who found strong asporin expression in cancer associated fibroblasts and its importance in coordinated invasion of gastric cancer. On the other hand, Maris et al. [25] have described the tumor suppressive potential of asporin in breast cancer. With respect to these contradictory results, the role of asporin in cancer progression and the tumor microenvironment deserves further investigation.

## RESULTS

### Asporin associates with better prognosis in low-grade tumors but not in high-grade breast cancer

**Figure 1:**
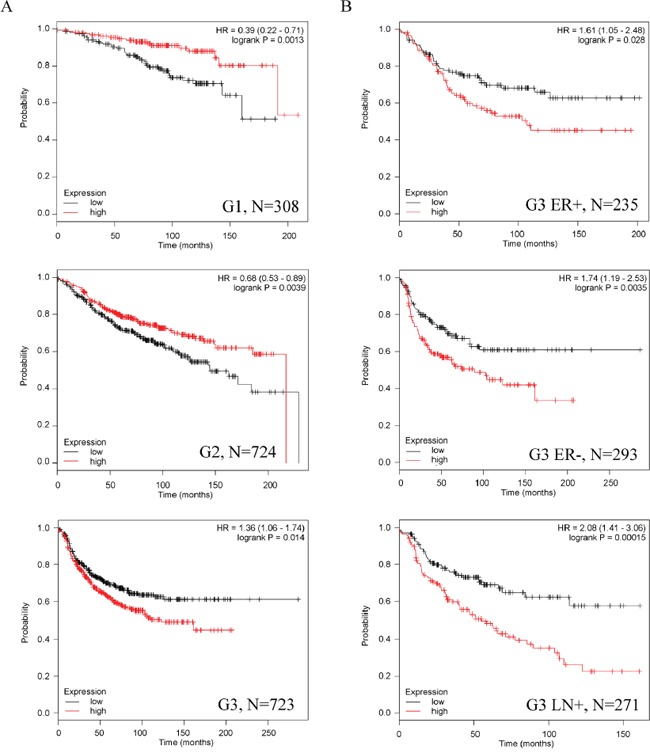
Asporin expression has different prognostic value in low-grade and advanced breast cancer **A.** High asporin expresion associates with significantly better relapse free survival (RFS) in patients with low-grade tumors but RFS is significantly worse in patients with grade 3 tumors. **B.** High asporin expression is associated with significantly worse RFS both in estrogen receptor positive and negative grade 3 tumors, in particular with metastasis to lymph nodes. HR, hazard ratio; N, number of patients in the KMPLOT analysis.

To acquire novel clues for the role of asporin in breast cancer we performed data mining using the Kaplan-Meier Plotter database comprising 4142 breast cancer patients. Detailed results on relapse free survival (RFS), overall survival and distant metastases free survival are summarized in Table 1 and Supplementary Table 1A. Although high asporin expression may serve as a good prognostic marker for tumor grades 1 and 2, the opposite can be observed in high-grade breast cancer (Figure 1A). High asporin expression is associated with significantly worse RFS both in estrogen receptor positive and negative grade 3 tumors, in particular with metastasis to lymph nodes (Figure 1B, Table 1). Asporin predicts good response to endocrine therapy in estrogen receptor positive breast cancer, in particular the luminal A molecular subtype. However, it is associated with worse RFS in the chemotherapy treated basal subtype or in the untreated luminal B subtype (Supplementary Table S1A).

**Table 1:** Prognostic value of high asporin expression in different breast cancer categories

subtype*	status**	prognostic value***	RFS	patients	status	prognostic value	OS	patients	status	prognostic value	DMFS	patients
any	any		0.43	3554	any	↑↑	0.046	1117	any		0.42	1609
	ER+	↑	0.067	1802	ER+	↑↑↑	0.0012	377	ER+	↑↑↑	0.0014	577
	ER-	↓↓	0.041	671	ER-		0.16	142	ER-		0.57	170
	Her2+	↓	0.081	168	Her2+		n.d.	28	Her2+		0.5	111
	Her2-	↑↑	0.048	756	Her2-		0.25	62	Her2-		0.18	82
	LN+		0.14	945	LN+		0.33	197	LN+		0.96	337
	LN-		0.61	1813	LN-	↑↑	0.034	425	LN-	↑↑	0.035	896
	G1	↑↑↑	0.0013	308	G1		0.13	135	G1		0.48	172
	G2	↑↑↑	0.0039	724	G2	↑↑↑	0.00053	287	G2	↑↑↑	0.0021	495
	G3	↓↓	0.014	723	G3		0.33	347	G3		0.21	391
	G3 ER+	↓↓	0.028	235	G3 ER+		0.58	96	G3 ER+		0.51	132
	G3 ER-	↓↓↓	0.0035	293	G3 ER-	↓	0.13	109	G3 ER-	↓	0.068	125
	G3 Her2+		0.67	87	G3 Her2+		n.d.	25	G3 Her2+		0.93	54
	G3 Her2-	↓↓↓	0.0025	239	G3 Her2-	↓	0.066	46	G3 Her2-		0.19	37
	G3 LN+	↓↓↓	0.00015	271	G3 LN+		0.32	122	G3 LN+		0.35	123
	G3 LN-		0.57	381	G3 LN-		0.38	160	G3 LN-		0.35	269
	G3 ER+ LN+	↓↓	0.041	117	G3 ER+ LN+		0.22	40	G3 ER+ LN+		0.9	48
	G3 ER+ LN-		0.85	114	G3 ER+ LN-		0.36	54	G3 ER+ LN-		0.75	81
	G3 ER- LN+	↓↓↓	0.0067	106	G3 ER- LN+		0.21	35	G3 ER- LN+		n.d.	27
	G3 ER- LN-		0.3	182	G3 ER- LN-		0.31	72	G3 ER- LN-		0.26	96
	G3 Her2- LN+	↓↓↓	0.0055	115	G3 Her2- LN+		0.18	30	G3 Her2- LN+		n.d.	14
	G3 Her2- LN-		0.48	123	G3 Her2- LN-		n.d.	16	G3 Her2- LN-		n.d.	23
luminal A	any		0.12	1764	any	↑↑	0.043	504	any	↑	0.074	918
	ER+	↑↑	0.027	1205	ER+	↑↑↑	0.0034	262	ER+	↑	0.048	419
	ER-		0.57	83	ER-		0.77	28	ER-		0.91	20
	LN+	↑↑	0.022	453	LN+	↑↑↑	0.00033	71	LN+		0.4	167
	LN-	↑↑	0.042	999	LN-		0.29	221	LN-	↑	0.065	546
	G3	↓	0.12	169	G3		0.5	73	G3		0.39	97
luminal B	any	↓↓	0.02	1002	any		0.52	320	any		0.77	361
	ER+	↓↓↓	0.0039	556	ER+		0.69	99	ER+		0.91	146
	ER-		0.88	128	ER-		0.28	22	ER-		0.81	28
	LN+	↓↓	0.017	275	LN+		0.5	43	LN+		0.61	87
	LN-		0.63	446	LN-	↓	0.091	80	LN-		0.38	171
	G3	↓	0.12	209	G3		0.24	96	G3		0.69	95
basal	any	↓	0.079	580	any		0.5	204	any		0.23	219
subtype	ER+		0.41	36	ER+		n.d.	14	ER+		n.d.	10
	ER-	↓↓	0.038	339	ER-		0.19	60	ER-		0.39	74
	LN+	↓↓	0.042	144	LN+		0.31	52	LN+		0.46	48
	LN-		0.69	291	LN-		0.67	96	LN-		0.74	134
	G3		0.17	263	G3		0.59	129	G3		0.57	136

* Molecular subtypes were derived from gene expression profiling at KMPLOT (http://kmplot.com/analysis/). With respect to the available Her2 status by FISH (see column status), the Her2 molecular subtype is not shown.

** Protein expression of estrogen receptor (ER+, ER-) may differ from mRNA expression which is used for molecular classification.

*** Arrows indicate positive (↑) or negative (↓) prognostic value of high asporin expression. Significance p<0.01, p<0.05 and trend p<0.15 are highlighted by three, two or one arrow, respectively.

n.d., not done for less than 30 patients; RFS, relapse free survival; OS, overall survival; DMFS, distant metastasis free survival.

Analysis of other cancer types showed significantly worse overall survival of ovarian and gastric cancer with high asporin expression. On the other hand, lung adenocarcinoma with high asporin expression had better outcome than patients with low asporin expression (Supplementary Table S1B).

### Identification of asporin positive cells *in-vitro* and antibody validation

**Figure 2:**
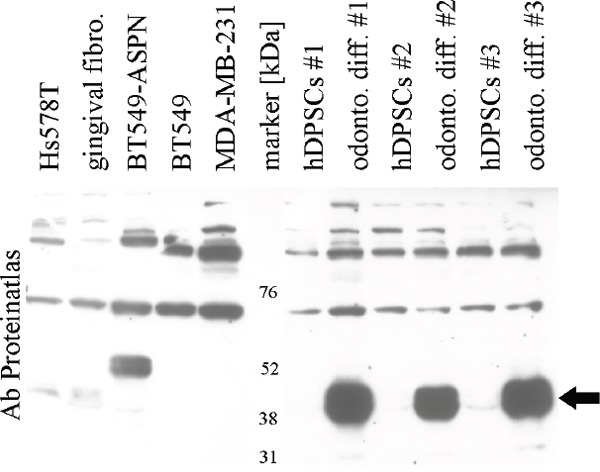
Validation of asporin antibody (Proteinatlas #HPA008435) in breast cancer cells, gingival fibroblasts and human dental pulp stem cells (hDPSCs) Black arrow indicates strong upregulation of asporin in hDPSCs after odontogenic differentiation. BT-549-ASPN with stable transfected asporin open reading frame sequence display a band with higher molecular weight (a similar band was observed also in MDA-MB-231-ASPN cells, data not shown). Validation of other antibodies is provided in Supplementary Figure S2.

We have searched public repositories (Gene Expression Omnibus and Array Express) and found Hs578T as the only breast cancer cell line with asporin expression (Supplementary Figure S1A), which we confirmed by qRT-PCR (see below). Western blot results with several antibodies were not clear in relation to mRNA expression of positive gingival fibroblasts and negative breast cancer cell lines BT-549 and MDA-MB-231 (Supplementary Figure S2, Supplementary Table S2). Given the urgent need for a reliable antibody for subsequent experiments we saught another positive control. Asporin plays an important role in collagen mineralisation [10] and it has recently been found to be strongly upregulated during odontogenic differentiation of human dental pulp stem cells (hDPSC) [26]. We reproduced this finding (three independent samples with mean ΔΔCt 4.93 which corresponds to approximately 30 fold mRNA upregulation) and western blot results were in concordance with the mRNA levels (Figure 2, Supplementary Figure S2). Presence of asporin in the indicated double-band was further confirmed by mass spectrometry (Supplementary Figure S3, Supplementary Table S3).

### Asporin is regulated by BMP4, serum-starvation and 3D cultivation in Hs578T cells

**Figure 3:**
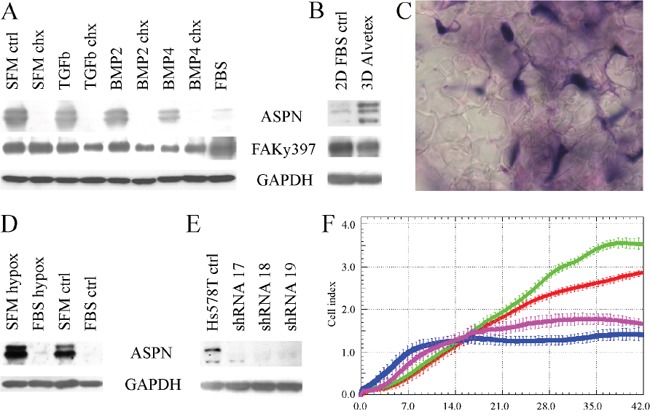
Glycoprotein asporin in Hs578T breast cancer cells **A.** Asporin is consistently upregulated by serum starvation (SFM-ctrl, serum-free medium control) and downregulated by two-day treatment with 100 ng/ml bone morphogenetic protein 4 (BMP4). Mild asporin downregulation by TGF-beta was not reproduced in other experiments. BMP4 also decreased phosphorylation of focal adhesion at tyrosine 397 (FAKy397). Inhibition of translation by cotreatment with 10 μg/ml cycloheximide led to downregulation of asporin but not of FAK. All treatments were performed in serum free media for 48 hours. **B.** Expression of asporin is upregulated by 12-day cultivation of Hs578T cells in Alvetex polystyrene scaffold (3D) in comparison to normal 2D conditions. **C.** Formallin-fixed paraffin-embedded Hs578T cells (stained with hematoxylin-eosin) in polystyrene Alvetex scaffold (transparent stellar shapes; magnification 1000x). **D.** Four-day cultivation of Hs578T cells in hypoxic conditions does not consistently change asporin expression. **E.** Expression of asporin is successfully downregulated with three different shRNAs. **F.** Invasion of Hs578T cells (parental cells are displayed in green, control cells with scrambled shRNA are in red) through collagen matrix is inhibited by shRNAs 17 and 19 (blue and purple, respectively). Similar inhibition was observed also for the shRNA 18 (data not shown). Reading of cell index was every 10 minutes up to 42 hours with the real time cell monitoring xCELLigence instrument (error bars indicate standard deviations from quadruplicate measurement). All experiments were performed at least three times and representative blots/chart are shown.

We have tested multiple treatments which could modify expression of asporin in Hs578T. Expression of asporin is increased by TGF-beta in chondrocytes [27, 28], however, we observed no consistent upregulation of asporin after TGF-beta (Figure 3A, 1.3 mean fold upregulation). Other members of the TGF-beta family, BMP2 and 4, have been reported to increase asporin levels in periodontal ligament cells [29]. In breast cancer Hs578T cells, BMP4 slightly downregulated asporin and phosphorylation of focal adhesion kinase (FAK) at tyrosine 397 (2.4 and 2.1 mean fold downregulation, respectively), while BMP2 did not cause any consistent changes (Figure 3A, 1.2 mean fold downregulation). BMP4 also downregulated the asporin mRNA level (three independent samples with mean ΔΔCt 3.77 which corresponds to approximately 14 fold mRNA downregulation).

Interestingly, asporin expression was markedly enhanced in serum free medium in comparison to a medium containing 10% FBS (Figure 3A and 3D, 22.5 mean fold protein upregulation; mean ΔΔCt 4.03 which corresponds to approximately 16 fold mRNA upregulation). Lack of nutrients may correspond to stress conditions for cancer cells *in vivo* where hypoxia also often occurs. We have tested cultivation of Hs578T in hypoxic conditions both in serum-free medium and with 10% serum, but we did not observe any consistent influence of hypoxia on asporin expression (Figure 3D, 1.1 mean fold upregulation in serum-free medium).

Asporin binds to collagen type I, inhibits its fibrillogenesis and may affect the extracellular matrix [10]. In this sense, we tested whether different 3D matrices can modulate asporin expression in Hs578T. The matrix of collagen type I (800 μg/ml) and Geltrex had no effect but 3D polystyrene Alvetex increased asporin expression (Figures 3B-3C; 10.0 mean fold protein upregulation; mean ΔΔCt 2.8 which corresponds to approximately 8 fold mRNA upregulation). Neither upregulation of asporin by serum starvation nor Alvetex matrix was associated with changes in phospho-FAK (Figure 3A-3B).

### Downregulation of asporin inhibits invasion of Hs578T through collagen matrix

In order to further test the importance of asporin expression in Hs578T we prepared three stable clones with shRNA against asporin. We confirmed downregulation of asporin expression (Figure 3E; mean ΔΔCt 5.3 which corresponds to approximately 40 fold mRNA downregulation) which did not affect proliferation, adhesion, migration or spheroid growth (data not shown). Importantly, downregulation of asporin inhibited invasion through the collagen type I matrix (Figure 3F).

We also generated stable clones of MDA-MB-231 and BT-459 cells with asporin ORF (open reading frame with myc and DKK tag) expression vectors. Expression of mRNA was comparable to Hs578T while immunoblotting detected a band with slower mobility (Figure 2; immunoblotting for MDA-MB-231 is not shown). Expression of asporin ORF did not affect proliferation, adhesion, migration or invasion of stable clones of BT-549 or MDA-MB-231 (data not shown). Interestingly, we were not able to prepare stable clones with expression vector with full asporin sequence (including 5′ and 3′UTR).

### Recombinant asporin enhances invasion through collagen type I matrix

**Figure 4:**
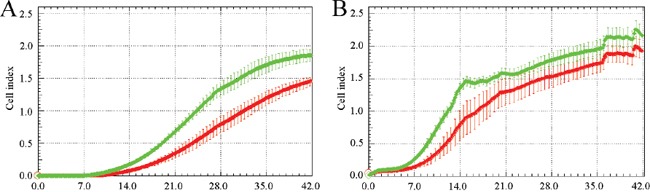
Recombinant asporin enhances invasion of MDA-MB-231 **A.** and BT-549 **B.** breast cancer cells through collagen matrix Both models were seeded at a density of 75000 cells per well into CIM plates coated by collagen stiffened in presence (green) or absence (red) of 10 nM recombinant asporin. The bottom chambers were filled with complete medium containing 10% FCS (fetal calf serum) as a chemoattractant. Reading of cell index was every 10 minutes up to 42 hours with the real time cell monitoring xCELLigence instrument (error bars indicate standard deviations from quadruplicate measurement). All experiments were performed at least three times and representative charts are shown.

Asporin binds to collagen type I, inhibits its fibrillogenesis and may affect extracellular matrix [7, 10]. Prof. Oldberg kindly provided us with the recombinant asporin which we used in invasion assays. First, we tested invasion of the three breast cancer cell lines mentioned above through various concentrations of collagen type I matrix (50, 200 and 800 μg/ml; data not shown) and selected a concentration of 200 μg/ml which was also used by Sodek et al. [30]. MDA-MB-231 and BT-549 cells invaded faster through the collagen matrix which was prepared with the recombinant asporin in comparison with the matrix without asporin (Figure 4A-4B). Invasion of Hs578T was comparable in both matrices (data not shown).

### RNA scope *in situ* hybridization detects asporin in cancer associated fibroblasts

**Figure 5:**
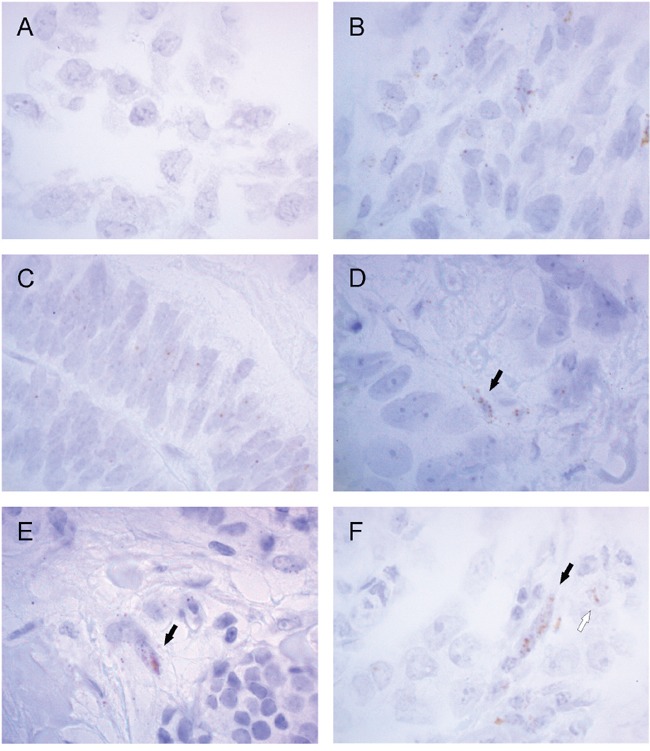
Asporin is detected by RNA scope *in situ* hybridization in invasive breast cancer Human dental pulp stem cells before **A.** and after **B.** odontogenic differentiation, as well as endometrium **C.**, were used as positive controls. Specific dot-like positivity was found in cancer associated fibroblasts both in ductal **D.** and lobular **E-F.** invasive carcinomas (black arrows). Asporin positivity was rarely observed also in cancer cells (F, white arrow). Magnification is 1000x while lower magnification (400x) of the same samples is provided in the Supplementary Figure S5.

We have identified asporin as a novel breast cancer related protein by laser microdissection and microarray analysis [12], in particular in invasive lobular carcinomas. However, asporin has been repeatedly reported to be expressed by cancer associated fibroblasts (CAFs) or other stromal cell types [15, 23, 24, 25]. Although laser microdissection is designed for single cell isolation we cannot exclude the possible capturing of adjacent cells, in particular in invasive lobular cancers which may grow in thin lines. As there is no reliable antibody for immunohistochemistry, we decided to use RNA scope technology with gene-specific probe pairs for detection of asporin in breast cancer tissues. As a positive control (Figure 5) we used endometrium (Supplementary Figure S4) and the above mentioned hDPSCs after odontogenic differentiation. Next, we hybridized 43 lobular and 7 ductal invasive breast cancer tissues. However, frequencies of specific dot-like signals were very low which precluded statistical analysis. Asporin dot-like positivity was found in CAFs both in ductal and lobular invasive carcinomas (Figure 5 and Supplementary Figure S5).

### Downregulation of asporin in cancer associated fibroblasts attenuates their coordinated invasion with breast cancer cells

**Figure 6:**
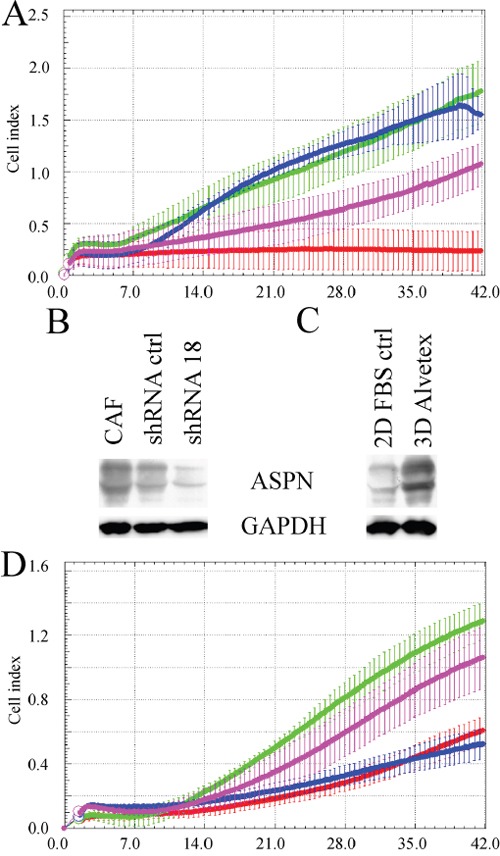
Invasion of breast cancer associated fibroblasts and T47D cells is attenuated by asporin downregulation **A.** Transwell experiment indicate coordinated invasion of cancer associated fibroblasts with T47D cells through extracellular matrix composed of collagen, matrigel and crosslinking ribose (70000 CAFs, green; 70000 T47D, red; CAFs + T47D, 35000 each, blue; 35000 CAFs, purple). Western blot analysis shows downregulation of asporin by shRNA **B.** and its upregulation upon 3D cultivation of CAFs in Alvetex polystyrene scaffold **C. D.** Downregulation of asporin attenuated invasion of CAFs and T47D cells (70000 CAFs shRNA ctrl, green; 70000 CAFs shRNA18, red; CAFs shRNA ctrl + T47D, 35000 each, purple; CAFs shRNA 18 + T47D, 35000 each, blue). All experiments were performed three times and representative blots/charts are shown.

We have tested three different CAFs from patients with grade 3 breast cancer (full details are in the Methods section) and selected those with the fastest invasion and proliferation for knock-down and co-culture experiments with T47D cells. This breast cancer cell line has low invasive potential alone and represents luminal B subtype [31] (please note poor RFS of luminal B patients with high asporin expression in Table 1). Transwell experiments indicated coordinated invasion of CAFs with T47D cells which was attenuated by asporin knock down in CAFs (Figure 6A) (Figure 6B and 6D).

Similarly to Hs578T, asporin expression was enhanced in CAFs by their 3D culture in Alvetex scaffold (Figure 6C). Analysis of aspartic acid (D) repeat length of asporin revealed that our breast CAFs have D13/D14 genotype which has recently been associated with poor metastasis-free survival of prostate cancer patients (Supplementary Figure S6) [32].

## DISCUSSION

The need for complex 3D culture models to unravel novel pathways and identify accurate biomarkers in breast cancer has been recently reviewed [33]. We have observed asporin upregulation during 3D culture of Hs578T cells in polystyrene scaffold Alvetex, but not in collagen or Geltrex matrices. Similarly, expression of tropomyosin kinase 2 was increased by 3D cultivation on collagen-coated polystyrene scaffold Alvetex [34] in comparison to 2D culture. Stiffness of the matrix is a major factor which could affect tumor progression [1, 35]. Polystyrene scaffold is stiffer than collagen or Geltrex matrices and may better simulate solid stress in tumors *in vivo*.

In contrast to chondrocytes or peridontal ligament cells [28, 29] we found no asporin modulation by TGF-beta or BMP2 in Hs578T cells. Nevertheless, asporin was slightly downregulated by BMP4. Similarly to BMP7, expression of BMP4 is low in invasive breast cancer and its signalling has been implicated in the maintenance of tumor dormancy [36–38]. Unexpectedly, we observed upregulation of asporin in serum-free cultivation. We speculate that this induction could lead to enhanced motility towards better sources of nutrients. Upregulation by starvation has also been reported for other proteins in cancer, e.g. glucose regulated chaperones [39, 40].

MDA-MB-231 and BT-549 cells invaded faster through collagen matrix which was prepared with the recombinant asporin in comparison to the matrix without asporin. This may be related to less dense matrix due to inhibition of collagen fibrillogenesis by asporin [10]. Invasion of Hs578T cells, which is accompanied by endogenous asporin production, was not further enhanced in the matrix with recombinant asporin. On the contrary, asporin downregulation decreased invasion of Hs578T cells through the collagen matrix. As shown by Satoyoshi et al. [24] asporin itself can promote invasion of both cancer cells and cancer associated fibroblast by interaction with CD44 and activation of Rac1. We have not observed any phenotypic change of BT-549 and MDA-MB-231 cells after stable transfection, however, asporin from ORF sequence may not reflect its native form.

We found asporin dot-like positivity mostly in cancer associated fibroblasts both in ductal and lobular invasive carcinomas which is in concordance with studies both from breast or other solid tumors such as prostate or gastric cancer [23, 24]. Cancer associated fibroblasts resemble myofibroblasts with smooth muscle actin which is also expressed in so called myoepithelial Hs578T [41]. Interestingly, Hs578T and SNB-75 cancer cells with asporin expression have the highest expression of smooth muscle actin (Supplementary Figure S1B). Asporin has also been reported to be expressed by gastric cancer cell lines and to enhance their oncogenic properties [42]. As asporin belongs to secreted proteins, regulation of its expression both in CAFs and cancer cell is relevant for alteration of the tumor microenvironment.

Maris et al. [25] have recently described the tumor suppressive potential of asporin in several breast cancer models. This may be true for a subset of breast cancer patients. However, *in silico* data mining on a large cohort of breast cancer patients showed that high asporin expression is associated with significantly worse RFS both in estrogen receptor positive and negative grade 3 tumors, even with metastasis to lymph nodes. Asporin was also associated with worse RFS in the chemotherapy treated basal subtype and in the untreated luminal B subtype. Van't Veer et al. [43] have recently assembled 72 breast cancer-related gene expression datasets, containing approximately 5700 samples altogether, where asporin is included in an extracellular matrix module comprising 58 genes. Importantly, asporin was found as one of the most upregulated genes in bone metastases of breast and prostate cancer [17, 44]. This is in accord with its primary function in skeletal development (mainly bones, cartilage and teeth), regulation of collagen fibrillogenesis and induction of mineralization [6, 7, 10, 45]. Hurley et al. [32] have recently described different metastatic potential of prostate cancer related to the polymorphism of aspartic acid (D)-repeat of asporin, which may also be important in breast cancer. D14 allele (heterozygous with D13 as well as in combination with any other allele) was associated with poor patient outcome, which is in line with decreased invasion of our breast CAFs upon asporin downregulation (genotype D13/D14). The unique asporin polyaspartate repeat binds calcium [10] and the D14 allele was also linked to predisposition for knee joint osteoarthritis whereas the D13 allele was more prevalent in healthy individuals [46]. The role of other alleles, such as D15 (homozygous in breast cancer cell line Hs578T) or D16 (heterozygous with D13 in bacteria used for production of recombinant asporin) awaits elucidation. Maris et al. [25] used transcript variant 1 with 13 aspartic acids (NM_017680.4) for both *in vitro* and *in vivo* experiments and therefore their results are in good concordance with the study of Hurley et al. [32] which decribed tumor suppressive effects of the D13 allele in prostate cancer.

In conclusion, we found that asporin can be downregulated by bone morphogenetic protein 4 in Hs578T cells and its upregulation may be facilited by serum-free cultivation or by three dimensional growth. Downregulation by shRNA inhibited invasion of Hs578T as well as of CAFs and T47D cells while invasion of asporin-negative MDA-MB-231 and BT549 breast cancer cells through collagen type I was enhanced by recombinant asporin. In contrast to these observations, tumor suppressive effects of asporin were published by others. Importantly, opposing effects of different asporin alleles have recently been reported in prostate cancer. Its dual role is also documented by our metaanalysis of publicly available data which found asporin to be associated with better prognosis in low-grade tumors but not in high-grade breast cancer. Besides other investigations, large scale analysis of aspartic acid (D)-repeat polymorphism will be needed for clarification of asporin dual role in progression of breast cancer.

## MATERIALS AND METHODS

### Cell culture

Authenticated and mycoplasma-free breast cancer cell lines were used for all experiments. Hs578t cells, obtained from ECACC (Salisbury, UK), were grown in Dulbecco's Modified Eagle's Medium (DMEM; Life Technologies, Carlsbad, CA) supplemented with 10 % foetal bovine serum (FBS, ThermoFisher, MA, USA), 1% penicillin/streptomycin (Life Technologies) and insulin (10 μg/ml) (Life Technologies). MDA-MB-231, BT-549 and T47D, obtained from American Type Culture Collection (Rockville, MD, USA), were cultivated in DMEM supplemented with 10% FBS and 1% penicillin/streptomycin.

Normal human gingival fibroblasts (kindly provided by prof. Jitka Ulrichova from Department of Medical Chemistry and Biochemistry at Palacky University Olomouc) were obtained from medically healthy donors who were clinically free of periodontal disease. Samples of gingiva were obtained from patients undergoing surgical removal of third molars at the Department of Oral and Maxillofacial Surgery (University Hospital, Olomouc). The tissue acquisition protocol adhered to the requirements of the local Ethics Committee. All patients had signed written informed consent.

The gingival tissues were washed three times in phosphate buffered saline (PBS) containing antibiotics (pamycon and colinomycin). The excised gingiva were cut into 1 mm pieces, plated in Petri dishes (10 cm diameter) and cultured in DMEM with 10% FBS and 1% penicillin/streptomycin. Explants were incubated in humidified atmosphere with 5 % CO_2_ at 37°C. Cells were fed weekly until the fibroblasts reached confluence. After 4-6 weeks cell cultures were trypsinized and transferred into 75 cm^2^ cultivation flasks. Cells were used between the 3 and 10 passages for experiments.

### Breast cancer associated fibroblasts

Isolation of primary breast cancer associated fibroblasts was performed according to modified protocol from Giannoni et al. [47, 48]. Tissue samples were obtained from patients undergoing surgical removal of breast cancer at Masaryk Memorial Institute (Brno, Czech Republic). The tissue acquisition protocol adhered to the requirements of the local Ethics Committee and all patients had signed written informed consent. Pieces of breast cancer tissue were minced and put on a 100 mm tissue culture dish. Sterile microscopic slide was put over the tissue pieces, slightly pushed, and overlaid with DMEM medium supplemented with 20% FBS, 2% penicillin/streptomycin, 100 μg/ml kanamycin (Serva, Germany), 2.5 μg/ml amphotericin B (ThermoFisher Scientific). Approximately 3 weeks after initial seeding of tissue pieces, the microscopic slide was removed and fibroblasts were further maintained as a regular cell culture. After approximately two weeks in culture, the cultivation medium was changed to DMEM with 10% FBS and 1% penicillin/streptomycin. To confirm homogeneity of isolated fibroblasts (denoted as BCAF3, BCAF4 and BCAF5), we performed FACS analysis of three selected surface fibroblast markers using BD FACSVerse flow cytometer. Cell suspension obtained by trypsinisation was stained with primary antibodies against FAP (human fibroblast activation protein alpha, MAB3715, R&D Systems), FSP (fibroblast surface protein, F4771, Sigma-Aldrich), followed by staining with secondary antibody Alexa Fluor 488 conjugated donkey anti-mouse IgG (H+L) (A21202, ThermoFisher Scientific); and primary antibody anti-fibroblast FITC (130-100-134, Miltenyi Biotec). Dead cells were excluded from the analysis using LIVE/DEAD Fixable Far Red Dead Cell Stain Kit (L10120, ThermoFisher Scientific). Analysis showed uniform positivity for all three markers and simultaneously, no positivity for non-fibroblast markers EpCAM (324220, BioLegend), CD31 (303104, BioLegend), and CD45 (304054, BioLegend) was detected.

We have isolated fibroblasts from three patients with invasive breast cancer, no special type (samples were denoted as BCAF3, BCAF4 and BCAF5) with the following clinicopatological parameters: BCAF3 - grade 3, pT2 (25 mm), pN0, M0, Her2 positive, ER 0%, PR 0%, AR 100%, Ki67 52%; BCAF4 - grade 3, pT2 (35mm), pN1a(3/15), M0, Her2 negative, all ER, PR and AR 100%, Ki67 30%; BCAF5 - grade 3, pT3 (55mm), pN0, Her2 negative, ER 100%, PR variable (0-3-80%), AR 80%, Ki67 55%. BCAF4 grew fastest and were more invasive than BCAF5 (data not shown; BCAF3 were not tested for invasion due to their slow proliferation). BCAF4 (passages from 9 to 11) were used as a model of cancer associated fibroblasts in all experiments.

Transient asporin knockdown in BCAF4 (specific sh18 and control scrambled shRNA; please see the sequences below) was performed with Viromer® transfection reagent (Lipocalyx, Hall, Germany) according to manufacturer protocol. Standard medium was changed after 12 hrs and one day later the cells were starved in serum free medium for 6 hrs before invasion experiments (see below).

### Human dental pulp stem cells and odontogenic differentiation

The isolation and culture of dental pulp stem cells (hDPSC) were performed according to protocol described previously [49]. Impacted wisdom teeth were obtained from healthy donors with informed consent at the Department of Dentistry under approved guidelines of the Ethical Committee of the Faculty Hospital in Hradec Kralove. For odontogenic differentiation, hDPSCs were cultured until confluence and then incubated with alpha-MEM supplemented with 10 mM beta-glycerolphosphate, 50 μM ascorbic acid and 0.1 μM dexamethasone for one week [26].

### Experimental treatments of Hs578T cells

Multiple treatments which could modify expression of asporin in Hs578T were tested. TGF β (transforming growth factor β) in concentration of 10 ng/ml, BMP2 and BMP4 (bone morphogenetic protein 2 and 4) in concentration 100 ng/ml were added to 24 hrs starved Hs578t cells in serum deprived media for 48 hrs. Additionally, each cytokine was combined with cycloheximide treatment (Sigma-Aldrich, Germany) in final concentration 10 μg/ml for the same period of time. Long term cultivation of Hs578t cells in presence of 1 μM dexamethasone (Sigma-Aldrich) was for three weeks. Expression of asporin was also monitored after 4 day cultivation of Hs578T cell under hypoxic conditions with 3% O_2_.

Cultivation of Hs578t in 3D was performed with polystyrene scaffold Alvetex® (Reinervate, UK), Geltrex® (Life Technologies) or collagen type I (Purecol®, Advanced BioMatrix, Carlsbad, CA) and lasted for 12 days with medium exchange every 4 days. Geltrex thin gel method was performed by mixing 500 μl of Geltrex with 1000 μl cultivating media followed by 1.5 hour incubation in 37°C. Collagen in a concentration of 800 μg/ml was diluted to 150 mM NaCl buffered with 20 mM Hepes (pH 7.4), and left to solidify in 37°C in cell culture incubator.

### Generation of stably transfected cell lines

For asporin overexpression MDA-MB-231 and BT-549 were transfected either with ASPN full length sequence (TrueClone, pCMV6-AC, Origene) or with open reading frame (TrueORF, pCMV6, Origene) sequence using Neon Transfection System (Life Technologies). Stable cell lines were selected with 0.5 mg/ml geneticin for 2 weeks and then kept under low selection pressure at 0.1 mg/ml geneticin.

For asporin knockdown Hs578t cells were similarly transfected with short hairpin RNAs (sh17 AGT TGA AAT ACC TCC AGA TAA TCT TCC TT, sh18 TCC AAC AGT GCC AAA GAT GAA GAA ATC TT or sh19 CGA GTA TGT GCT CCT ATT ATT CCT GGC TT in pRS plasmid, OriGene) and selected with 1 μg/ml puromycin. Both overexpression and knockdown of ASPN were checked by RT-PCR and western blotting. Control cells were transfected with empty pRS plasmid or scrambled shRNA (GCA CTA CCA GAC CTA ACT CAG ATA GTA CT; Origene #TR30012).

### Adhesion, proliferation and migration assays

Experiments were carried out using xCELLigence Real-Time Cell Analyzer (RTCA) DP instrument (Roche diagnostic, GmbH, Germany). For adhesion assay 40 000 cells were seeded on E-plate 16 surface and increasing cell index was monitored every 30 seconds for 4 hrs, followed by recording cell proliferation for additional 64 hrs.

For migration monitoring, fully confluent cell population in a standard cultivation medium supplemented with 10 μg/ml mitomycin C (Roche) was scratched by pipette tip. Migrating cell populations were monitored every second hour for 24 hrs. Experiments were repeated at least three times.

### Invasion assays

Invasion assays were carried out using xCELLigence Real-Time Cell Analyzer (RTCA) DP instrument (Roche). Parental breast cancer cell lines (MDA-MB-231, BT-549 and Hs578t) were tested with collagen I matrix prepared with or without recombinant asporin (kind gift from Professor Åke Oldberg, University of Lund). Bottom side of CIM plate inserts (Roche/Acea, CA, USA) was pre-coated with 30 μl of cooled 200 μg/ml collagen I (Purecol®, Advanced BioMatrix, Carlsbad, CA) prepared as described by Kalamajski et al. [10]. Resulting aqueous phase was pipetted off after 30 minutes leaving a thin collagen film. The upper side of the inserts was coated with 30 μl of cooled collagen I with or without 10 nM recombinant asporin and the solution was incubated at 37°C for 6 hrs. The lower chamber of the CIM plate was filled with 170 μl 10% FCS DMEM as a chemoattractant. Background signal was measured after addition of 30 μl of serum-free media per well followed by 1 hour of incubation at 37°C. Cells which were starved in medium without serum for 6 hrs were harvested and seeded in amount of 75000 cells per insert well. Cell index was monitored every 10 minutes for 42 hrs.

For stably transfected cells (MDA-MB-231, BT-549 and Hs578t) the invasion barrier was prepared from 200 μg/ml pure collagen I (collagen G1, Matrix BioScience GmbH Germany) and CIM plate was coated as described above. Collagen solution in the upper part of wells was incubated for 1 hour at 37°C and then 75000 starved cells were added. Increasing cell index was recorded every 10 minutes for 24 hrs. All experiments were repeated at least three times.

Modified protocol was used for CAFs and T47D cells. The upper side of the inserts was coated with 30 μl of mixture of 200 μg/ml neutralized collagen I (Advanced BioMatrix, Carlsbad, CA), Matrigel® (200 μg/ml; Corning, MA, USA) and 15 mM D-ribose (Sigma-Aldrich) and then incubated at 37°C for 1 hr [50]. Lower chamber of the CIM plate was filled with 170 μl 10% FCS DMEM as a chemoattractant. Collagen-Matrigel-ribose clot was overlaid by 30 μl of serum-free media. Assembled coated CIM plates were left at 37°C in the incubator for 1 hr followed by reading background signal. Starved cells were harvested and seeded in amount of 70000 cells per insert well or 35000 per cell line in co-culture experiments. Cell index was monitored every 10 minutes for 42 hrs. Experiments were repeated three times.

### Spheroid assay

Spheroid cell culture was performed by the liquid overlay method [51]. Briefly, all cell models were seeded at 10 000 cells on convex face of 1.5% agarose LE (Promega, WI, USA) in DMEM. The plate was then spun at 1000 rpm for 15 minutes to induce cell aggregation and subsequently each well was overlayed by 100 μl of standard cultivation media [52]. Formation of mammospheres was recorded after 4 days.

### *In situ* hybridization by RNAscope and immunohistochemistry

Archival tissue samples of 50 invasive breast cancer, among them 43 invasive lobular (ILC) and 7 invasive ductal carcinomas (IDC) were obtained from patients between years 1999 and 2006. We have previously found high levels of asporin in ILC than IDC [12], therefore, ILC cases were specifically selected to reach sufficient sample size. The study was approved by the Ethics Committee of the Faculty of Medicine and Dentistry. Control cells from *in vitro* culture were homogeneously distributed in an agarose gel matrix, creating an artificial tissue, and then formalin-fixed and paraffin-embedded [53]. These controls were used also for immunohistochemistry validation (Supplementary Table S2), however, the results didn't reflect the mRNA levels of asporin in any method tested (antigen retrieval by boiling either in a microwave for 20 min in citrate buffer pH6 or in a water bath in EDTA pH9; visualization by DAB chromogen and Dual Link secondary antibody, Dako) [13].

*In situ* hybridization for asporin RNA was performed using the RNAscope® 2.0 kit according to the manufacturer's manual (Advanced Cell Diagnostics, USA; [54]. Briefly, 5 μm formalin-fixed, paraffin-embedded tissue sections were deparaffinized in xylene and pre-treated with heat (15 minutes, boiling in water bath) and protease (undiluted protease solution, 30 minutes, 40°C, DAKO hybridizer). Length of the main hybridization step was increased from 2 to 3 hrs (40°C). Then signal amplification system which included hybridization with preamplifier, amplifier and label probes, and subsequently chromogenic detection with DAB (3,3′-diaminobenzidine) was used. Positive staining was identified as brown punctate dots in the cell. Negative control probes (DapB) and positive control probes (POLR2A) were also included for each examined slide.

With respect to the RNA scope technology, several aspects should be commented upon. The frequency of dot-like positivity was low, while other types of positivities occurred, for example in areas with inflammation or rare homogenous nuclear positivity in negative control. The results may also be affected by variability of patient samples. Although all samples were fixed and embedded in our pathology laboratory according to standard protocols, we observed differences in signals with RNA scope positive control. Dreyer et al. [55] compared RNA scope with DNA *in situ* hybridization and PCR detection of HPV infections. Surprisingly, RNA scope signals occurred even after digestion with RNase. In our opinion, RNA scope technology may support results obtained by other methods but is not a robust enough method for routine use as yet.

### Mass spectrometry

Cells were lysed and 200 μg of proteins was separated on SDS-PAGE as described below. Gel was coomassie stained and relevant molecular weight bands were excised, destained, reduced by 50 mM Tris(2-carboxyethyl) phosphine hydrochloride (Sigma-Aldrich), alkylated by 50 mM Iodoacetamide (Sigma-Aldrich) and digested with proteomic grade trypsin (Promega) at 37°C overnight. Mass spectrometric analysis was performed on an Orbitrap Fusion (ThermoFisher Scientific) instrument fitted with a Proxeon Easy-Spray ionization source, coupled to an Ultimate 3000 RSLCnano chromatograph. Ten microliter of sample was loaded on a μ-Precolumn C18 PepMap 100 (300 μm x 5 mm, 5 μm, 100 Ȧ pore size) desalting column (ThermoFisher Scientific) “in-line” with a PepMap RSLC (75 μm x 50 cm, 3 μm, 100 Ȧ pore size) analytical column (ThermoFisher Scientific) heated at 35°C. The peptides were subsequently separated on the analytical column by ramping the organic phase from 5% to 35% during a total run time of 165 minutes. The aqueous and organic mobile phases were 0.1% formic acid diluted in water or acetonitrile, respectively. There were two parallel experiments running simultaneously on mass spectrometer. The first one was single FTMS scan with resolution was set to 120,000 and precursor ions were scanned across an m/z range of 400- 1600. The second experiment was targeted MS2 with HCD collision and Orbitrap detector. Resolution was set to 30,000 and HCD collision energy to 35%. The transitions of most suitable peptides were designed and resulting spectra were evaluated in Skyline-daily 2.6.1.6899 software [56].

### Production of recombinant asporin

One milliliter aliquot of frozen bacterial culture containing the plasmid carrying the gene for asporin was thawed and suspended in 100 ml of LB medium (10 g NaCl, 10 g tryptone, 5 g yeast extract per liter) with kanamycin (75 mg/L) and incubated overnight on a heated rotation shaker (37°C) [10]. The next day this starting culture was added to 1 liter of fresh LB medium containing kanamycin. The culture was again incubated on rotation shaker until reaching the sufficient density for the induction (O.D._600_=0.6). Expression of the recombinant asporin was induced by Isopropyl-β-D-thiogalacto-pyranoside (IPTG, final concentration 1 mM). Bacteria were incubated for another 5 hours and then harvested by the centrifugation. Bacterial pellet was resuspended in denaturating lysis buffer (8 M urea buffered by 50mM Tris, pH 8), sonicated (10×10 s, 2 min intervals) and centrifugated (10000g, 10 min, room temperature). The obtained supernatant was used for protein purification using the metaloaffinity chromatografy with Ni-NTA agarose. Washing and elution was performed using buffers with decreasing pH (8 M urea, pH 6.3; 5.9 and 4.5). Glutathion-S-transferase recombinant asporin (H00054829-P01, Abnova, Taiwan) was purchased for quantification of asporin by mass spectrometry (Supplementary Figure S3).

### RNA isolation, reverse transcription and quantitative PCR

Total RNA isolation was performed by the RNeasy Mini Kit (Qiagen), quantified by Nanodrop, pretreated with Dnase I (Invitrogen, Carlsbad, CA, USA) and reverse transcribed with random hexamers and SuperScript III Reverse Transcriptase (Invitrogen). The real-time polymerase chain reaction reactions were performed using specific primers and probes (ASPN forward 5′GGG TGA CGG TGT TCC ATA TC 3′, reverse 5′TTG GTG GTA AGC CTT TAG GAA 3′, probe 5′ BHQ1-TTG CAG AAG CAA AAC TGA CC-FAM 3′; TBP forward 5′CAC GAA CCA CGG CAC TGA TT 3′, reverse 5′TTT TCT TGC TGC CAG TCT GGA C 3′, probe 5′ BHQ1-TCT TCA CTC TTG GCT CCT GTG CAC A-HEX 3′) and Probes Master Mix for 50 cycles of denaturation, annealing and extension (95–60–72°C each for 20 s) at the LightCycler 480 instrument (Roche). Relative quantification was carried out according to the ΔΔCt method using TBP as a reference gene [57].

### Protein extraction and western blot analysis

Cells were harvested into RIPA buffer (50 mM Tris HCl pH 8.0; 150 mM NaCl; 1% NP-40 0.5% sodium deoxycholate; 0.1% SDS) supplemented with protease/phosphatase inhibitor cocktail (Roche). Twenty micrograms of whole cell lysate were separated by electrophoresis in 10% Bis-Tris polyacrylamide gel followed by blotting to nitrocellulose membrane. Non-specific binding sites were blocked by incubating the blots for 2 hrs at room temperature with 5% (w/v) non-fat dry milk in PBS. Blots were incubated overnight with primary antibodies at the following concentrations: anti-ASPN (1:1000; # HPA008435; Sigma-Aldrich, Protein Atlas; plus other antibodies specified in Supplementary Table S2), anti-FAK pY397 (1:500, Life Technologies), and anti-GAPDH (1:25000; Sigma-Aldrich) was used as a loading control. Secondary antibodies were as follows anti-rabbit IgG, HRP-linked Antibody (#7074) and Anti-mouse IgG, HRP-linked Antibody (#7076), both purchased from Cell Signaling Technology, MA, USA. Signal detection was performed with Dura/ Femto ECL western blotting substrate (ThermoFisher Scientific). Analysis of optical density was performed using ImageJ software.

### In silico data mining

Expression of asporin in cancer cell lines was checked either in Gene Expression Omnibus database at the National Center for Biotechnology Information or in ArrayExpress database at the European Bioinformatics Institute. The prognostic and predictive value of asporin (ASPN; 219087_at) was further evaluated by Kaplan-Meier Plotter (KMPLOT) [58], where all settings were left at default values (database version 2014; breast cancer analysis was performed from 8^th^ to 10^th^ September 2015; other cancer types were analysed on 23^rd^ February 2016).

### Genotyping of the asporin D-repeat polymorphism

The aspartic acid (D) - repeat polymorphism located in the N-terminal region of the ASPN gene was evaluated according to Hurley et al. [32]. Briefly, PCR products (fluorescent forward primer 5′ 6-FAM–ATT CCT GGC TTT GTG CTC TG and non-labeled reverse primer 5′ TGG CTT CTT GGC TCT CTT GT) were mixed with GeneScan™ 500 TAMRA™ Size Standard and Hi-Di™ Formamide (Applied Biosystems, Foster City, CA, USA) and denatured 3 min at 95°C. Then, it was separated on ABI PRISM® 3130 Genetic Analyzer and obtained data were analysed and visualised using GeneScan® Analysis Software (Applied Biosystems). Genotypes of BCAF3, 4 and 5 were D13/D14, D13/D14 and D13/D15, respectively.

Genotype of three samples (BCAF4, Hs578T and plasmid DNA from *E.coli* producing recombinant asporin; Supplementary Figure S6) was confirmed by Sanger sequencing. PCR was performed with the same primers (without FAM tag in the forward primer) using HotStarTaq Master Mix (Quiagen, Hilden, Germany). Products were purified by QIAquick PCR Purification kit (Qiagen) and used as a template for sequencing PCR which was performed using BigDye Terminator v1.1 Cycle Sequencing kit (Applied Biosystems). PCR products of this second PCR were purified by BigDye XTerminator™ Purification Kit (Applied Biosystems) and then separated by capillary electrophoresis on ABI PRISM® 3100 Genetic Analyzer (Applied Biosystems). Obtained data were analysed using Sequencing Analysis Software™ and visualised with GeneMapper® Software (Applied Biosystems). Whole procedure was performed according to the manufacturer protocol.

## SUPPLEMENTARY FIGURES AND TABLES






