# Supplementary material for: The dual role of asporin in breast cancer progression
Source: Oncotarget. 2016 Jul 7;7(32):52045–60. doi: 10.18632/oncotarget.10471 (PMC5239534; doi:10.18632/oncotarget.10471)
Supplement: Supplementary file 1 [file oncotarget-07-52045-s001.pdf]

The dual role of asporin in breast cancer progression

SUPPLEMENTARY FIGURES AND TABLES

A

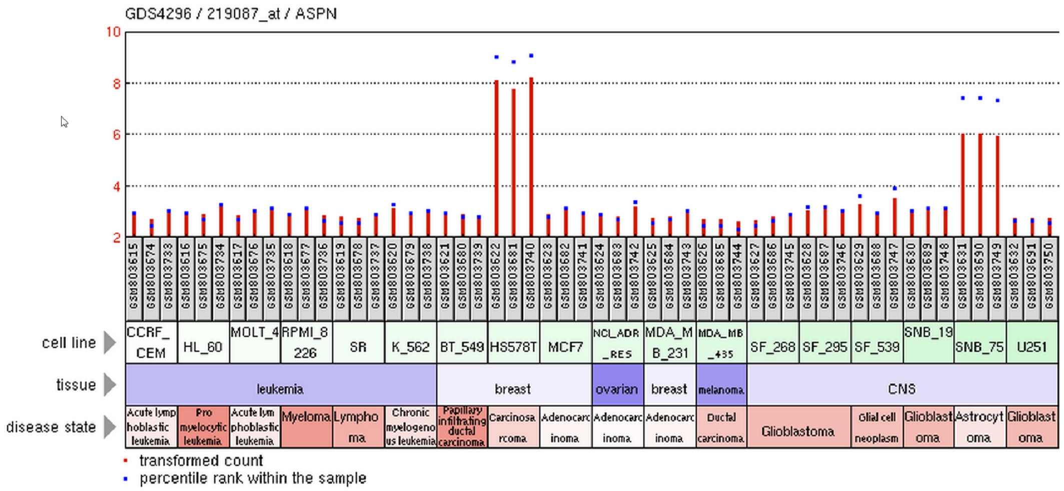

B

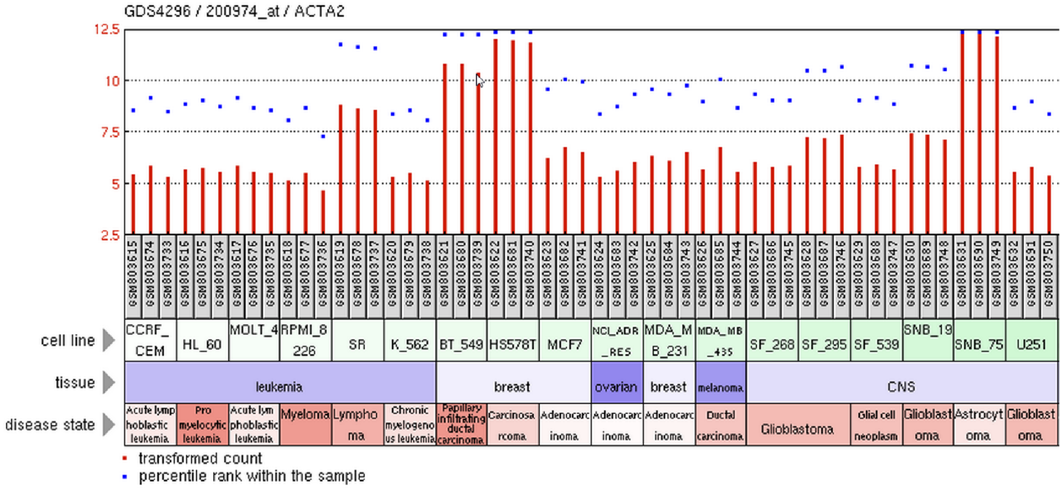

Supplementary Figure S1: Microarray analysis in NCI-60 cancer cell line panel (GDS4296 in GEO Database at the National Center for Biotechnology Information). A. Histogram with two positive cell lines is shown while all other cell lines were negative (full histogram can be easily found in the GEO Database). Hs578T breast cancer cell line has higher signal also in GSE5720, GSE15026 and GSE24717. Other cell lines with asporin mRNA expression can be found in Cancer Cell Line Encyclopedia (Supplementary Table S4). **B.** Expression of smooth muscle actin (gene name ACTA2) is highest in the asporin positive cell lines Hs578T and SNB-75.

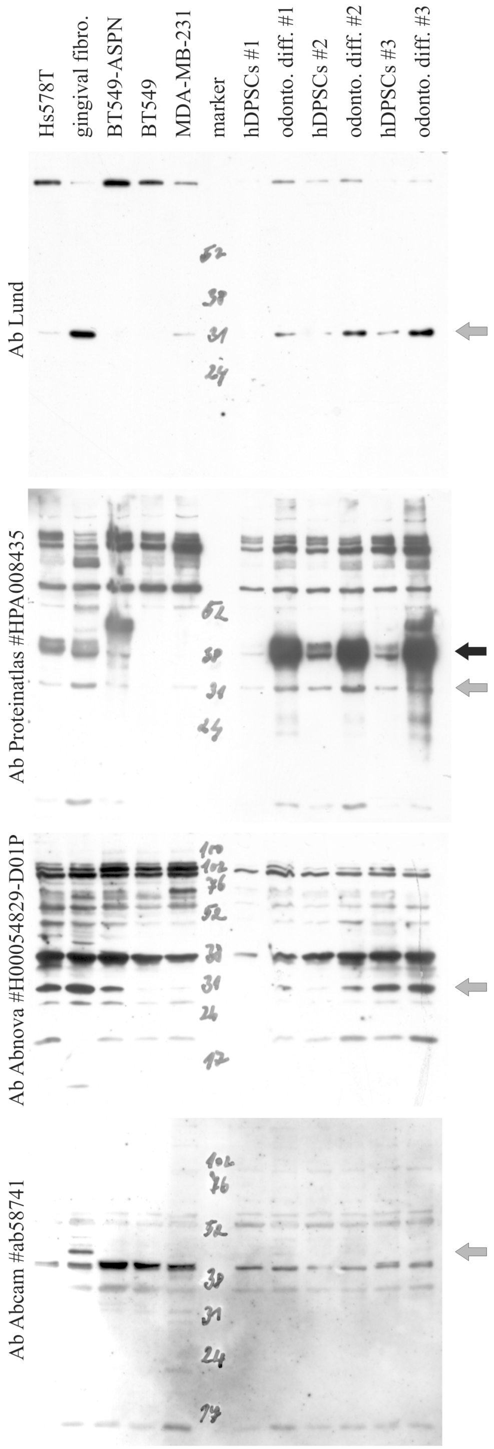

Supplementary Figure S2: Validation of the indicated antibodies (antibody Lund was kindly provided by prof. Oldberg; see also Supplementary Table S2). Grey arrows indicate bands which partially correspond with RT-PCR results. Black arrow is commented in the main Results section.

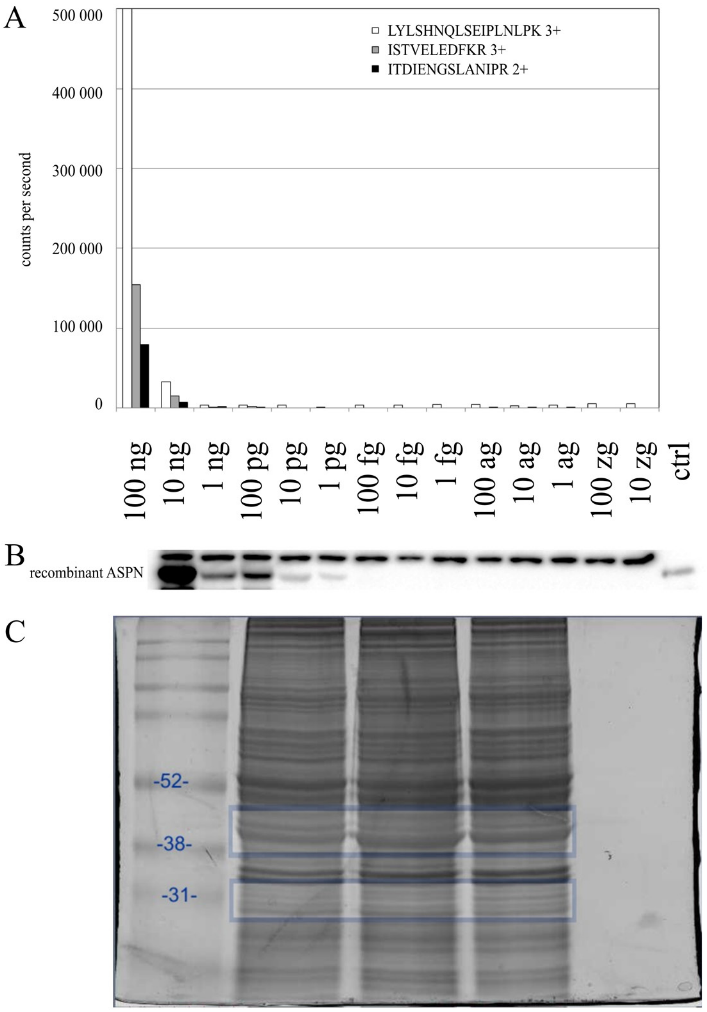

Supplementary Figure S3: Mass spectrometric analysis of asporin. Dilution series was prepared by spiking recombinant asporin in indicated amount into 10 ul lysate of HeLa cells. Mass spectrometric analysis detected a sample with 10 ng recombinant asporin **A.,** while western blotting with the Proteinatlas antibody #HPA008435 detected a sample with 1 pg of asporin **B.** The sample with 100 ng was not analysed by western blotting due to expected very high signal. One ng of recombinant asporin (without HeLa lysate) was loaded as a control directly for electrophoresis (ctrl; molecular weight of the recombinant asporin is 70 kDa due to the fusion with glutathion S-transferase). Experiment was repeated three times, representative image is shown. Supplementary Figure S3 **C.** displays polyacrylamide gel with loading of 200 µg protein of hDPSCs after odontogenic differentiation (three independent samples). The gel was cut at the indicated molecular weights and analysed by mass spectrometry (see Supplementary Table S3). A similar gel was prepared for BT549-ASPN.

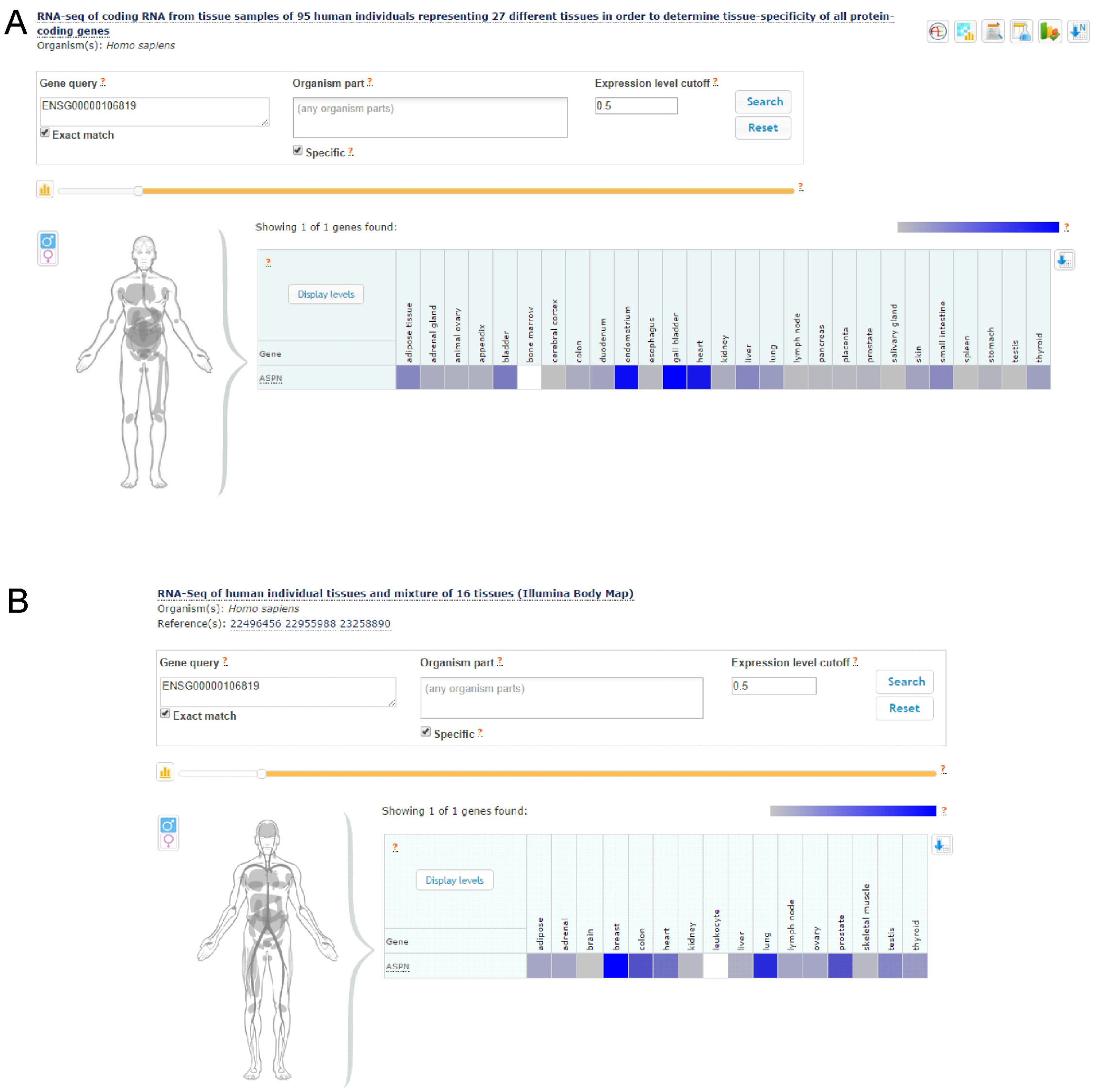

Supplementary Figure S4: Expression of asporin by RNA sequencing in different human tissues (Expression Atlas of ArrayExpress database at the European Bioinformatics Institute). The highest expression was detected in endometrium, gall bladder, heart **A.** and breast (**B.,** Illumina Body Map).

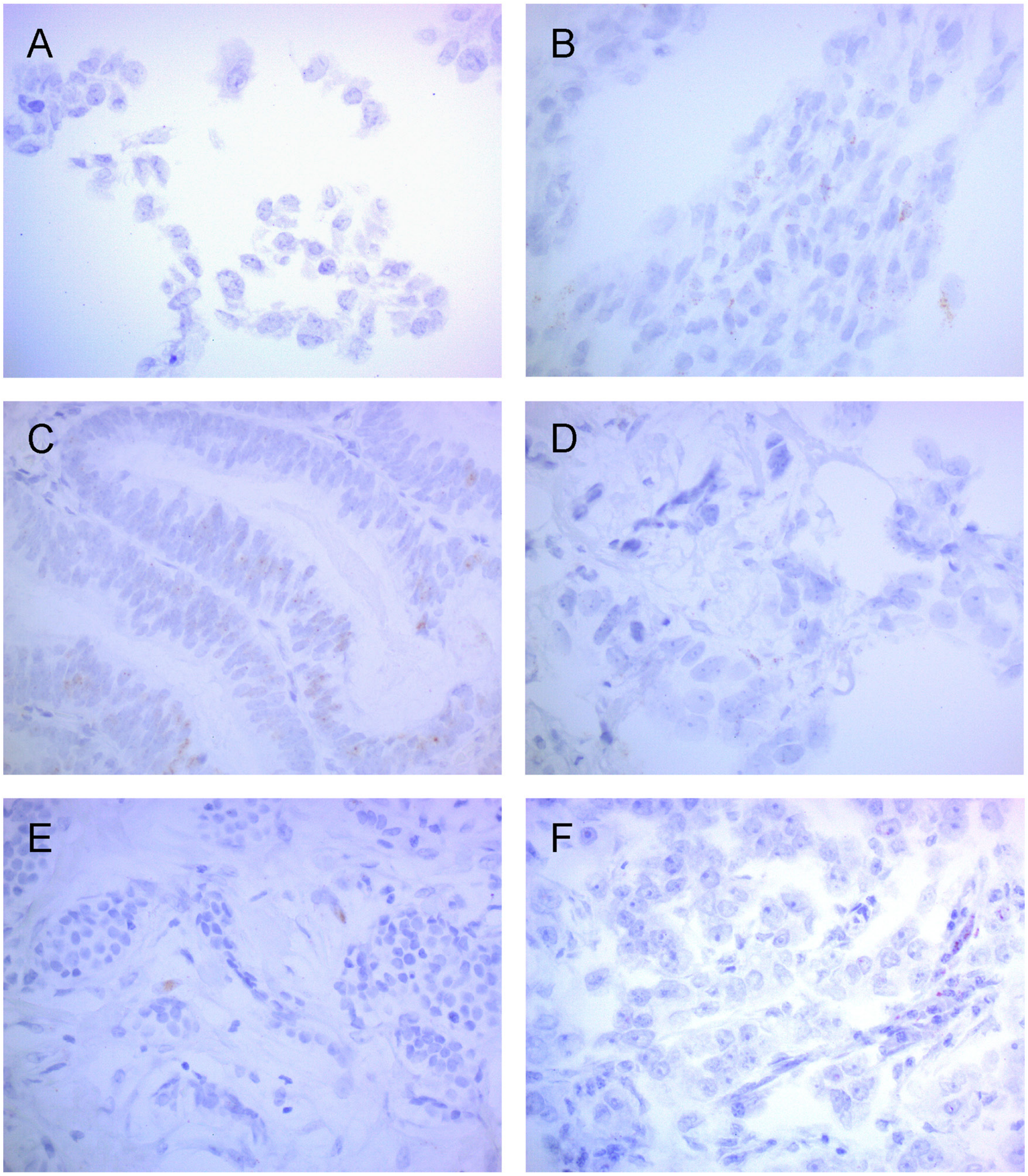

Supplementary Figure S5: Expression of asporin by RNA scope *in situ* hybridization. The same samples as in the Figure 4 are shown at lower magnification (400x).

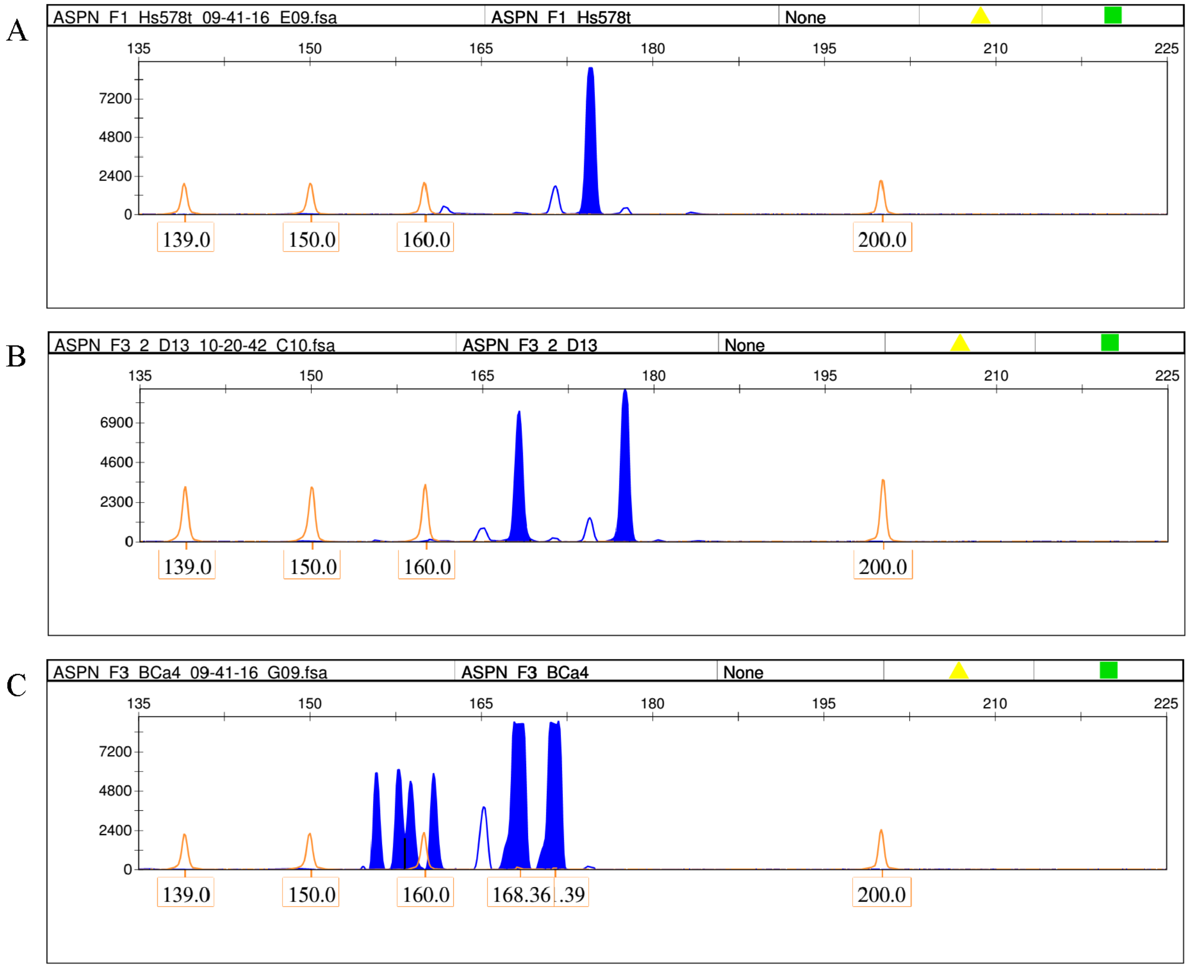

Supplementary Figure S6: Genotyping of asporin D-repeat polymorphism. Hs578T cells contain D15 allele **A.,** while breast cancer associated fibroblasts have D13/D14 genotype **C.** Besides expected D13 allele, the asporin producing *E.coli* contained also D16 allele **B.**, which was confirmed by Sanger sequencing (along with Hs578T cells and CAFs). Axes Y and X display fluorescent intensity and capillary electrophoresis mobility, respectively (please see Material and Methods for further details).

Supplementary Table S1A: Prognostic value of high asporin expression in various treatments of breast cancer subtypes

Supplementary Table S1B: Prognostic value of high asporin expression in ovarian, lung and gastric cancer

See Supplementary File 1

Supplementary Table S2: Overview of asporin antibodies

Supplier	producer	host	cat.no.	immunogen	tested in this study	published article [1]
Sigma-Aldrich	proteinatlas.org	rabbit	HPA008435	IPLNLPKSLAELRIHENKVKKIQKDTFKGMNALHV LEMSANPLDNNGIEPGAFEGVTVFHIRIAEAKLTS VPKGLPPTLLELHLDYNKISTVELEDFKRYKELQRL GLGNNKITDIENGSLAN	Figure 1, Suppl. Figure 2, IHC not shown	Satoyoshi et al. 2014, Maris et al. 2015, Hurley et al. 2016
Abcam/ GenWay	not specified	rabbit	ab58741/ GWB-6BA1C3	ISTVELEDFKRYKELQRLGLGNNKITDIENGSLANIP RVREIHLENNKL	Suppl. Figure 2, IHC not shown	Gruber et al. 2009 (GenWay), Lee et al. 2011 (Abcam catalog # not specified)
Prof. Oldberg	Lund group	rabbit	in-house	expressed from cDNA	Suppl. Figure 2, IHC not shown	
Abcam/ Everest-Biotech	not specified	goat	ab31303/ EB06670	IHENKVKKIQKDT	data not shown	Ho et al. 2010, Hurng et al. 2011
Abnova	Abnova	rabbit	H00054829-D01P	full recombinant protein	Suppl. Figure 2	
our antibody	Dr. Vojtesek	rabbit	in-house	TVELEDFKRYKELQR	data not shown	
our antibody	Dr. Vojtesek	rabbit	in-house	HLENNKLKKIPSGLPE	data not shown	
not available	Yamada group	rabbit	in-house	EPRSHFFPFD	not tested	Yamada et al. 2006
Abcam	not specified	rabbit	ab154404 and ab75317	YGLILNNNKLTKIHPKAFLTTKKLRRLYLSHNQLSEIP LNLPKSLAELRIHENKVKKIQKDTFKGMNALHVLEM SANPLDNNGIEPGAFEGVT and synthetic peptide derived from the C-terminal domain, respectively	not tested	
Sigma-Aldrich	proteinatlas.org	rabbit	HPA024230	EYVLLLFLALCSAKPFFSPSHIALKNMMLKDMEDTDDD DDDDDDDDDDDEDNSLFPTREPRSHFFPFDLFPMCPF GCQCYSRVVHCSDLGLTSVPTNIPFDTRMLDLQNNKI KEIKENDFKGLTSLYGLILNNNKLTKIHPKA	not tested	

[1] References not included in the main article: Gruber et al. Asporin, a susceptibility gene in osteoarthritis, is expressed at higher levels in the more degenerate human intervertebral disc. Arthritis Research & Therapy 2009, 11: R47; Ho et al. The biomechanical characteristics of the bone-periodontal ligament-cementum complex. Biomaterials 2010; 31:6635-46; Hurng et al. Discontinuities in the human bone-PDL-cementum complex. Biomaterials 2011; 32:7106-17.
IHC, immunohistochemistry.

Supplementary Table S3: Mass-spectometric intensities (counts per second) of asporin peptides in samples cut from a gel with loading of 200 µg protein (Figure S3C)

sample	Pep1	Pep2	Pep3
Odonto.diff#1 40 kDa	3000	1500	3000
Odonto.diff#1 30 kDa	0	0	0
Odonto.diff#2 40 kDa	2000	1500	2000
Odonto.diff#2 30 kDa	0	0	0
Odonto.diff#3 40 kDa	3000	1000	2000
Odonto.diff#3 30 kDa	0	0	0
BT549-ASPN 50 kDa	2000	2000	2500
BT549-ASPN 40 kDa	0	0	0

Pep1, thrice charged LYLSHNQLSEIPLNLPK; Pep2, twice charged ISTVELEDFKR; Pep3, thrice charged ISTVELEDFKR. Specific transitions were validated by the analysis of the recombinant asporin.

Supplementary Table S4: Expression of asporin by microarray analysis in Cancer Cell Line Encyclopedia (http://www.broadinstitute.org/ccle/home)

See Supplementary File 2
