# Supplementary material for: The dual role of asporin in breast cancer progression
Source: Oncotarget. 2016 Jul 7;7(32):52045–60. doi: 10.18632/oncotarget.10471 (PMC5239534; doi:10.18632/oncotarget.10471)
Supplement: Supplementary file 2 [file oncotarget-07-52045-s002.docx]

Supplementary Table 1A. Prognostic value of high asporin expression in various treatments of breast cancer subtypes

| treatment* | status** | prognostic value*** | RFS | patients | status** | prognostic value | OS | patients | status** | prognostic value | DMFS | patients |
| --- | --- | --- | --- | --- | --- | --- | --- | --- | --- | --- | --- | --- |
| untreated | any |  | 0.44 | 1000 | any | ↑ | 0.14 | 375 | any | ↑ | 0.079 | 533 |
| patients | luminal A |  | 0.17 | 517 | luminal A |  | 0.16 | 215 | luminal A | ↑ | 0.096 | 310 |
|  | luminal B | ↓ | 0.051 | 271 | luminal B |  | 0.18 | 89 | luminal B |  | 0.8 | 125 |
|  | Her2 | ↓ | 0.15 | 41 | Her2 |  | n.d. | 17 | Her2 |  | n.d. | 26 |
|  | basal |  | 0.67 | 171 | basal |  | 0.66 | 54 | basal |  | 0.41 | 72 |
|  | G3 |  | 0.23 | 207 | G3 |  | 0.3 | 110 | G3 | ↓ | 0.081 | 153 |
|  | ER+ |  | 0.48 | 500 | ER+ |  | 0.24 | 248 | ER+ | ↑↑ | 0.021 | 224 |
|  | ER+ luminal A | | 0.23 | 324 | ER+ luminal A | | 0.47 | 178 | ER+ luminal A | | 0.6 | 160 |
|  | ER+ luminal B | ↓↓↓ | 0.0076 | 157 | ER+ luminal B | | 0.51 | 58 | ER+ luminal B | | 0.7 | 54 |
|  | ER+ G3 |  | 0.97 | 55 | ER+ G3 | ↓ | 0.09 | 46 | ER+ G3 |  | 0.96 | 50 |
|  | ER- |  | 0.32 | 253 | ER- |  | 0.8 | 86 | ER- |  | 0.19 | 101 |
|  | ER- Her2 | ↓ | 0.09 | 32 | ER- Her2 |  | n.d. | 16 | ER- Her2 |  | n.d. | 19 |
|  | ER- basal |  | 0.85 | 138 | ER- basal |  | 0.48 | 43 | ER- basal |  | 0.21 | 46 |
|  | ER- G3 |  | 0.32 | 113 | ER- G3 |  | 0.53 | 61 | ER- G3 |  | 0.14 | 67 |
| chemo- | any |  | 0.22 | 274 | any |  | 0.31 | 69 | any |  | 0.69 | 65 |
| therapy | ER+ |  | n.d. | 23 | ER+ |  | n.d. | 20 | ER+ |  | n.d. | 1 |
|  | ER- |  | 0.21 | 211 | ER- |  | n.d. | 9 | ER- |  | 0.78 | 64 |
|  | basal | ↓↓ | 0.048 | 125 | basal |  | n.d. | 17 | basal |  | n.d. | 24 |
|  | G3 | ↓ | 0.15 | 165 | G3 | ↓ | 0.11 | 44 | G3 |  | 0.51 | 55 |
|  | G3 LN+ | ↓↓ | 0.048 | 80 | G3 LN+ |  | n.d. | 14 | G3 LN+ |  | n.d. | 27 |
| endocrine | any | ↑↑ | 0.029 | 849 | any | ↑↑↑ | 0.00001 | 128 | any |  | 0.73 | 513 |
| treated | ER+ |  | 0.24 | 725 | ER+ | ↑↑↑ | 0.00052 | 66 | ER+ | ↑ | 0.099 | 293 |
|  | ER+ luminal A | | 0.21 | 505 | ER+ luminal A | ↑↑↑ | 0.000067 | 40 | ER+ luminal A | ↑ | 0.1 | 230 |
|  | ER+ luminal B | | 0.49 | 209 | ER+ luminal B | | 0.59 | 26 | ER+ luminal B | | 0.55 | 61 |
|  | G3 |  | 0.77 | 125 | G3 | ↑ | 0.072 | 39 | G3 |  | 0.51 | 72 |
|  | LN+ |  | 0.42 | 233 | LN+ | ↑↑↑ | 0.0029 | 47 | LN+ |  | 0.73 | 206 |
|  | G3 LN+ | ↓↓ | 0.049 | 36 | G3 LN+ |  | n.d. | 10 | G3 LN+ |  | n.d. | 29 |
|  | ER+ G3 |  | 0.16 | 82 | ER+ G3 |  | n.d. | 13 | ER+ G3 |  | 0.92 | 55 |

* Molecular subtypes were derived from gene expression profiling at KMPLOT (http://kmplot.com/analysis/).

** Protein expression of estrogen receptor (ER+, ER-) may differ from mRNA expression which is used for molecular classification. In contrast to the Table 1, Her2 subtype is derived from expression profiling.

*** Arrows indicate positive (↑) or negative (↓) prognostic value of high asporin expression. Significance p< 0.01, p<0.05 and trend p<0.15 are highlighted by three, two or one arrow, respectively.

n.d., not done for less than 30 patients; RFS, relapse free survival; OS, overall survival; DMFS, distant metastasis free survival

Supplementary Table 1B. Prognostic value of high asporin expression in ovarian, lung and gastric cancer

| Ovarian | prognostic value | PFS | patients | Ovarian | prognostic value | OS | patients | Ovarian | prognostic value | PPS | patients |
| --- | --- | --- | --- | --- | --- | --- | --- | --- | --- | --- | --- |
| any | ↓↓↓ | 0.0001 | 1306 | any | ↓↓ | 0.0047 | 1582 | any | ↓↓ | 0.006 | 708 |
| G1 |  | n.d. | 37 | G1 | ↓ | 0.054 | 56 | G1 |  | n.d. | 14 |
| G2 | ↓ | 0.1 | 247 | G2 |  | 0.2 | 315 | G2 | ↓ | 0.11 | 138 |
| G3 | ↓↓↓ | 0.00031 | 790 | G3 | ↓ | 0.053 | 968 | G3 | ↓↓ | 0.027 | 513 |
| G4 |  | n.d. | 1 | G4 |  | n.d. | 2 | G4 |  | n.d. | 1 |
| PFS, progression free survival; OS, overall survival; PPS, post progression survival | | | | | | |  |  |  |  |  |
|  |  |  |  |  |  |  |  |  |  |  |  |
| Lung | prognostic value | FP | patients | Lung | prognostic value | OS | patients | Lung | prognostic value | PPS | patients |
| any |  | 0.36 | 982 | any | ↑↑↑ | 0.0001 | 1926 | any | ↑↑ | 0.0017 | 344 |
| adenocarcinoma | ↑↑ | 0.034 | 461 | adenocarcinoma | ↑↑↑ | 0.0001 | 720 | adenocarcinoma | ↑ | 0.051 | 125 |
| squamous cell carcinoma | | 0.27 | 141 | squamous cell carcinoma | | 0.41 | 524 | squamous cell carcinoma | | n.d. | 20 |
| G1 |  | 0.49 | 140 | G1 |  | 0.92 | 201 | G1 |  | 0.8 | 79 |
| G2 |  | 0.084 | 165 | G2 | ↑ | 0.12 | 310 | G2 |  | 0.36 | 89 |
| G3 | ↓ | 0.13 | 51 | G3 |  | 0.997 | 77 | G3 |  | n.d. | 24 |
| FP, first progression; OS, overall survival; PPS, post progression survival | | | | | |  |  |  |  |  |  |
|  |  |  |  |  |  |  |  |  |  |  |  |
| Gastric | prognostic value | FP | patients | Gastric | prognostic value | OS | patients |  |  |  |  |
| any | ↓↓↓ | 0.00098 | 641 | any | ↓↓↓ | 0.00022 | 876 |  |  |  |  |
| G1 |  | n.d. | 5 | G1 | ↓↓ | 0.037 | 32 |  |  |  |  |
| G2 | ↓↓ | 0.04 | 67 | G2 | ↓↓ | 0.028 | 67 |  |  |  |  |
| G3 | ↓↓ | 0.019 | 121 | G3 | ↓↓ | 0.039 | 165 |  |  |  |  |
| FP, first progression; OS, overall survival | | | |  |  |  |  |  |  |  |  |
| Please see Supplementary Table 1a for explanation of "prognostic value". | | | | | |  |  |  |  |  |  |
