# Supplementary material for: The dual role of asporin in breast cancer progression
Source: Oncotarget. 2016 Jul 7;7(32):52045–60. doi: 10.18632/oncotarget.10471 (PMC5239534; doi:10.18632/oncotarget.10471)
Supplement: Supplementary file 3 [file oncotarget-07-52045-s003.docx]

Supplementary Table 4. Expression of asporin by microarray analysis in Cancer Cell Line Encyclopedia (http://www.broadinstitute.org/ccle/home).

| rank ^1^ | 54829_at | cell line | Pubmed abstracts ^2^ | source, # ^3^ | comments from the source (e.g. ATCC) |
| --- | --- | --- | --- | --- | --- |
| 1 | 12.31037 | TE125T SOFT TISSUE | 0 | NBL/ATCC CRL-7945 | rhabdomyosarcoma, morphology fibroblast |
| 2 | 9.725762 | **DKMG CENTRAL NERVOUS SYSTEM** | **2** | **DSMZ, ACC 277** | **glioma, fibroblast-like** |
| 3 | 9.575321 | HS737T BONE | 0 | NBL/ATCC CRL-7473 | giant cell sarcoma, morphology fibroblast |
| 4 | 9.539002 | HS819T BONE | 0 | NBL/ATCC CRL-7891 | chondrosarcoma, morphology fibroblast |
| 5 | 9.087705 | HS600T SKIN | 0 | NBL/ATCC CRL-7360 | melanoma |
| 6 | 8.746542 | HS742T BREAST | 0 | NBL/ATCC CRL-7482 | scirrhous adenocarcinoma, morphology fibroblast |
| 7 | 8.595847 | **BJHTERT SKIN** | **2** | **ATCC, CRL-2522** | **normal fibroblast, immortalized** |
| 8 | 8.525169 | HS840T UPPER AERODIGESTIVE TRACT | 0 | NBL/ATCC CRL-7573 | papilloma, morphology fibroblast |
| 9 | 8.439707 | HS821T BONE | 0 | NBL/ATCC CRL-7554 | giant cell sarcoma, morphology fibroblast |
| 10 | 8.274899 | HS839T SKIN | 0 | NBL/ATCC CRL-7572 | melanoma, morphology fibroblast |
| 11 | 7.787109 | **OUMS27 BONE** | **5** | **JCRB, IFO50488** | **chondrosarcoma** |
| 12 | 7.760104 | T173 BONE | 0 | NBL/ATCC CRL-7943 | osteosarcoma, morphology fibroblast |
| 13 | 7.740823 | **KNS81 CENTRAL NERVOUS SYSTEM** | **1** | **JCRB, IFO50359** | **glioma, fibroblast-like** |
| 14 | 7.72326 | TE159T SOFT TISSUE | 0 | NBL/ATCC CRL-7752 | rhabdomyosarcoma, morphology fibroblast |
| 15 | 7.567781 | HS618T LUNG | 0 | NBL/ATCC CRL-7380 | adenocarcinoma, morphology fibroblast |
| 16 | 7.565485 | HS281T BREAST | 0 | NBL/ATCC CRL-7227 | adenocarcinoma, morphology fibroblast |
| 17 | 7.505275 | HS751T HAEMATOPOIETIC AND LYMPHOID TISSUE | 0 | NBL/ATCC CRL-7488 | cell line no more available |
| 18 | 7.501762 | HS739T BREAST | 0 | NBL/ATCC CRL-7222 | adenocarcinoma, morphology fibroblast |
| 19 | 7.487105 | HS895T SKIN | 0 | ATCC, CRL-7637 | melanoma, morphology fibroblast; Unlike other cell lines in the NBL Collection, this item has been fully accessioned by ATCC . |
| 20 | 7.387764 | HS616T HAEMATOPOIETIC AND LYMPHOID TISSUE | 0 | ATCC, CRL-7378 | Hodgkin´s lymphoma; Unlike other cell lines in the NBL Collection, this item has been fully accessioned by ATCC. |

^1^ Out of 1036, seventy eight cell lines have value higher than 5 RFU (relative fluorescence unit; 54829_at asporin probe set ID at Affymetrix Human Genome U133 Plus PM Array [Brainarray Version 13, HTHGU133PlusPM_Hs_ENTREZG]). Breast cancer cell line Hs578T was ranked 62nd.

^2^ Only 4 out of 20 top-ranked cell lines have been included in scientific articles (according to the abstract search at NCBI)

^3^ ATCC (www.atcc.org), DSMZ (www.dsmz.de), JCBR (http://cellbank.nibio.go.jp/english/), NBL - comment from ATCC: "One purpose of the NBL collection was to produce and distribute early passage primary cultures of cells from various clinical materials. Many of these cultures, unlike most others available from ATCC, are mixed populations. NBL lines from tumors, for example, may consist of mixtures of stromal and cancer cells in which the former cell type predominates."
